# Link Between Diet Quality and Lifestyle Factors Among Young Adults in Saudi Arabia

**DOI:** 10.3390/healthcare14081010

**Published:** 2026-04-12

**Authors:** Nahla Mohammed Bawazeer, Abeer Salman Alzaben, Huny M. Bakry, Raseel Mohammed Alrashidi, Khulood Sami Hussein

**Affiliations:** 1 Department of Health Sciences, College of Health and Rehabilitation Sciences, Princess Nourah Bint Abdulrahman University, P.O. Box 84428, Riyadh 11671, Saudi Arabia; 2 Department of Clinical Physiology, Faculty of Medicine, King Abdulaziz University, P.O. Box 80200, Jeddah 21589, Saudi Arabia

**Keywords:** diet quality, short healthy eating index, physical activity, sleep, young adults, Saudi Arabia

## Abstract

**Background/Objectives**: Poor diet quality is common among young Saudi adults, characterised by high fast-food intake and low fruit and vegetable consumption. This study investigated the association between diet quality and lifestyle factors using a validated short Healthy Eating Index (sHEI). **Methods**: This study adopted a cross-sectional design, gathering responses through a self-completed online questionnaire. Demographic data were collected. Diet quality was assessed using the Arabic version of the sHEI, physical activity was evaluated with the Arabic short form of the International Physical Activity Questionnaire (IPAQ), and sleep quality was measured using the Athens Insomnia Scale (AIS). Saudi residents aged 18–25 years were eligible; pregnant or lactating individuals and those with chronic conditions affecting dietary intake were excluded. **Results**: Among 478 participants (mean age 21.1 ± 1.9 years), 88.1% were female, 24.7% were overweight or obese, and half reported poor diet quality. Moderation scores were slightly higher (54.2% high), whereas adequacy scores were nearly equal (49.8% high and 50.2% low). Most participants reported low physical-activity levels (78.5%) and poor sleep quality (55.2%). Sleep quality was significantly associated with all diet quality measures, increasing the odds of good total sHEI (OR = 1.74, *p* = 0.003), adequacy (OR = 1.49, *p* = 0.034), and moderation (OR = 1.54, *p* = 0.021). **Conclusions**: Sleep quality is significantly associated with diet quality among young Saudi adults. While body mass index and physical activity showed no significant associations, improving sleep quality may promote healthier dietary behaviours. Future studies should explore pathways linking sleep and diet.

## 1. Introduction

Diet quality has become an important concept in nutritional research, particularly in epidemiology, as it provides a comprehensive measure of how well dietary patterns meet nutrient requirements and support health [1]. It reflects the balance between food groups and nutrients consumed, emphasising variety and nutrient-dense choices while limiting unhealthy components such as added sugars and saturated fats [2]. Strong evidence has demonstrated that higher diet quality lowers the likelihood of developing chronic conditions, such as obesity, type 2 diabetes, and cardiovascular disease, whereas poor diet quality is linked to greater susceptibility to these outcomes [3]. Consequently, diet quality scores are increasingly used to assess eating behaviours and predict long-term health risks, including morbidity and mortality [2].

A widely used tool for measuring diet quality is the Healthy Eating Index (HEI), developed to assess adherence to dietary recommendations. To make this approach more practical, a shorter version—the Short Healthy Eating Index (sHEI)—was developed and validated by Colby et al. (2020) and Shams-White et al. (2023) [3,4]. The sHEI is a 22-item questionnaire that evaluates key components of dietary intake, such as fruits, vegetables, dairy, added sugars, sugar-sweetened beverages, and calcium, offering a reliable yet user-friendly tool for researchers and participants alike [3]. However, its application in young adults in Saudi Arabia remains limited, particularly in relation to lifestyle correlates.

In Saudi Arabia, diets are commonly high in fats, sugar, and salt, while the intake of nutrient-rich foods such as fruits, vegetables, dairy, nuts, and fish remains low [5,6]. These trends are particularly evident among adolescents and young adults, where fast food consumption and energy-dense, nutrient-poor diets are widespread [7,8,9,10]. Many young people also report skipping balanced meals in favour of sugary beverages and snacks [7]. Such behaviours are strongly associated with rising rates of obesity and diet-related chronic diseases in Saudi Arabia, with recent figures showing that approximately 23% Saudi adults are classified as obese [11], 23.1% are diagnosed with diabetes [12], and 9.2% are affected by hypertension [13].

Young adulthood (18–30 years) represents a critical life stage during which long-term eating patterns and lifestyle behaviours are formed. Research indicates that this age group undergoes a critical transitional phase influenced by social and environmental factors that significantly impact dietary behaviours [14]. Existing evidence suggests that many young adults in Saudi Arabia, especially female university students, do not meet the recommended intake of fruits and vegetables, and that the overall diet quality in this age group remains suboptimal [15]. Establishing healthy dietary practices at this stage is particularly important, as it influences both productivity and long-term health [15,16].

Among lifestyle behaviours, physical inactivity and poor sleep patterns have been identified as prevalent and modifiable concerns among young adults in Saudi Arabia, with potential implications for dietary patterns [17]. Lifestyle factors such as sedentary behaviour and inadequate sleep further influence diet quality [18,19,20]. Evidence indicates that regular physical activity and sufficient sleep are positively associated with better diet quality [19,20]. In addition, body mass index (BMI) is commonly examined as a health indicator reflecting the cumulative effects of diet and lifestyle behaviours [21]. Moreover, these lifestyle factors are strongly associated with obesity risk [22]. Conversely, adopting healthier lifestyle practices, including improved diet, regular exercise, and sufficient sleep, may enhance adherence to dietary recommendations and overall diet quality.

Despite this evidence, lifestyle factors are often examined in isolation, with limited research exploring the combined association with overall diet quality in young adults, particularly in Saudi Arabia. Research in this population remains limited, especially studies integrating validated measures such as the sHEI with multiple lifestyle and health indicators. Consequently, the associations between lifestyle behaviours such as sleep quality, physical activity, and BMI as a health indicator, and overall diet quality have not been adequately examined among young Saudi adults.

To address this gap, the present study utilised the validated sHEI, which was previously applied to this age group in Saudi Arabia by Alzaben and Bawazeer (2025) [23], to assess diet quality and its relationship with key lifestyle and health indicators. We hypothesised that diet quality among young Saudi adults is suboptimal and that higher diet quality is positively associated with healthier sleep patterns and other favourable lifestyle behaviours.

## 2. Materials and Methods

### 2.1. Study Design and Subjects

A cross-sectional study was conducted between August 2022 and August 2023 using a self-administered online questionnaire. The study targeted young adults living in the major regions of Saudi Arabia. Eligible participants were Saudi adults aged 18–25 years currently living in Saudi Arabia. Individuals who were pregnant or lactating, or who had chronic conditions likely to influence dietary intake, such as food allergies, coeliac disease, diabetes mellitus, or cardiovascular disease, were excluded.

Ethical approval was granted by the Institutional Review Board of Princess Nourah bint Abdulrahman University (Approval No. 22-0935, dated 7 November 2022). All participants provided informed consent, and measures were taken to ensure confidentiality and anonymity.

### 2.2. Sample-Size Calculation and Technique

The target population comprised young Saudi adults, estimated at 3,919,150 individuals based on data from the General Authority for Statistics (2021) [24]. The minimum required sample size was calculated using a 95% confidence level (Z = 1.96), an assumed prevalence of 50% (*p* = 0.5), and a 5% margin of error (c = 0.05), resulting in a minimum sample size of 385 participants. Ultimately, 478 participants were included to reduce sampling variability. However, given the use of non-probability snowball sampling, increasing sample size does not ensure representativeness, and thus the generalizability of the findings remains limited. Recruitment was initiated through multiple initial participants and disseminated across various social networks to broaden reach.

### 2.3. Study Tool

A self-administered online questionnaire was developed using REDCap. The survey link was distributed to eligible individuals, who were encouraged to share it with others through various social media platforms. Participants provided demographic information, including age, sex, education level, employment status, marital status, and region of residence in Saudi Arabia. Height (cm) and weight (kg) were recorded, and BMI (kg/m^2^) was calculated and categorised as underweight (<18.5), normal weight (18.5–24.9), overweight (25.0–29.9), and obese (≥30.0).

### 2.4. Short Healthy Eating Index (sHEI)

sHEI, a tool originally developed and validated by Colby et al. (2020) [3] was used to assess diet quality. It was later translated and culturally adapted for the Saudi population and demonstrated high reliability and validity by Alzaben and Bawazeer (2025) [23]. The overall diet quality score was derived from the sum of 13 components. Adequacy scores represented dietary elements encouraged for sufficient intake to promote health and were calculated from nine components: total fruits, whole fruits, total vegetables, greens and beans, whole grains, dairy, total protein, seafood and plant protein, and fatty acids [25]. Moderation scores captured dietary factors that should be limited to reduce adverse health effects and were determined from four components: refined grains, sodium, added sugars, and saturated fats [25]. Participants were categorised as having good or poor diet quality based on whether their scores were above or below the median.

### 2.5. Physical Activity

Physical activity was measured using the official Arabic short form of the International Physical Activity Questionnaire (IPAQ) [26]. This tool assesses vigorous and moderate activities, walking, and sedentary behaviour over the previous seven days, covering multiple domains including occupational, transport, household and leisure time activities. Activity levels were categorised as low, moderate, or high according to Al-Hazzaa’s (2007) guidelines [27]. Metabolic equivalent (MET-minutes/week) scores were calculated using the following standard formulas: walking = 3.3 × minutes × days; moderate = 4.0 × minutes × days; and vigorous = 8.0 × minutes × days.

### 2.6. Sleep Quality

Sleep quality was assessed using the Athens Insomnia Scale (AIS), which evaluates sleep difficulty based on the International Classification of Diseases criteria [28]. The scale consists of eight components: sleep induction, awakening during the night, final awakening, total sleep duration, overall sleep quality, well-being during the day, functioning during the day, and sleepiness during the day. The scale was translated into Arabic by a team of clinical nutrition students and subsequently validated by bilingual linguistics experts in public health. The internal consistency of the tool was acceptable (Cronbach’s α = 0.769). Each component is scored from 1 to 4, with higher scores indicating more severe problems. The total AIS score (range: 8–32) was calculated by summing the eight component scores. A total AIS score above the median (14) was considered indicative of poor sleep quality.

### 2.7. Statistical Analysis

Data were entered and analysed using the Statistical Package for the Social Sciences (SPSS) version 25. Categorical variables were presented as numbers (n) and percentages (%), while continuous variables were expressed as mean ± standard deviation (SD). Spearman’s correlation was used to assess the relationships between the adequacy, moderation, and total sHEI scores and lifestyle factors, while associations between them were examined using the chi-square test. Binomial logistic regression was performed to predict the probability of an outcome related to the sHEI scores based on lifestyle factors. Spearman’s correlation and logistic regression were selected as they are robust to unequal group sizes, thereby providing reliable estimates. A *p*-value ≤ 0.05 was considered statistically significant.

## 3. Results

A total of 478 participants were included in the study. Table 1 shows their sociodemographic characteristics. The mean age was 21.1 ± 1.9 years; 54.6% were aged 20–22 years, and 88.1% were female. The majority were unmarried (93.5%), 71.6% were university-educated, and 82.8% were students. Regarding the region of residence, 56.5% lived in the central region and 22.3% in the western region of Saudi Arabia.

Table 2 presents participants’ diet quality and related factors. Overall diet quality was evenly distributed, with 50% classified as having good and 50% poor diet quality scores. Moderation scores were slightly higher, with 54.2% of the participants achieving high moderation scores, whereas adequacy scores were nearly equal (49.8% high; 50.2% low). Most participants reported low physical activity levels (78.5%), with only 11.7% reporting high activity levels and 9.8% moderate activity, indicating generally sedentary behaviour among participants. Sleep quality was poor among 55.2% of participants. suggesting that more than half experienced inadequate sleep. Regarding BMI, the majority were of normal weight (56.7%), while 18.6% were underweight, and 24.7% were overweight or obese.

Table 3 shows the Spearman correlations between diet quality components (adequacy, moderation, and total sHEI scores) and lifestyle factors. BMI was negatively correlated with adequacy (r = −0.090, *p* = 0.048), indicating that higher BMI was associated with slightly lower adequacy scores. Sleep quality was positively correlated with moderation (r = 0.159, *p* < 0.001) and total sHEI scores (r = 0.139, *p* = 0.002), suggesting that better sleep was linked to higher diet quality. Age and physical activity were not significantly correlated with any diet quality component.

Table 4 presents the associations between diet quality components (adequacy, moderation, and total sHEI scores) and lifestyle factors using the chi-square test. No significant associations were observed between BMI, physical activity, and any diet quality component. However, sleep quality was significantly associated with both moderation (*p* = 0.02) and total sHEI scores (*p* = 0.005), with participants reporting good sleep being more likely to have higher diet quality scores. Adequacy scores were not significantly associated with sleep quality (*p* = 0.67).

Table 5 shows the results of binomial logistic regression analyses examining the associations of lifestyle factors with total sHEI, adequacy, and moderation scores. Sleep quality was significantly associated with all diet quality measures; a one-unit increase in sleep quality was associated with higher odds of good total sHEI (OR = 1.74, *p* = 0.003), adequacy (OR = 1.49, *p* = 0.03), and moderation (OR = 1.54, *p* = 0.02). In contrast, BMI and physical activity were not significantly associated with diet quality components.

## 4. Discussion

This study investigated how diet quality is linked to lifestyle factors and associated indicators—namely, sleep quality, physical activity, and BMI—among young adults in Saudi Arabia. Among our findings, sleep quality was significantly associated with overall diet quality, including both adequacy and moderation, whereas no significant correlation was found between physical activity, BMI, and diet quality. These outcomes suggest that sleep may play an important role in improving dietary behaviour in young adults, regardless of BMI and physical activity levels. Similarly, prior research in Saudi Arabia found that poor lifestyle habits, including nutritionally deficient diets and low levels of physical activity, contribute to unhealthy weight status and poor diet quality [22], highlighting the importance of including multiple lifestyle factors in the evaluation of the diet–behaviour relationship.

The significant correlation between sleep quality and diet found in the present study corroborates the growing body of evidence indicating that sleep quality may influence food choices, appetite regulation, and eating behaviours. In a systematic review of 29 studies, Godos et al. (2021) reported a consistent correlation between better diet quality and improved sleep quality [29]. Diets rich in plant-based foods, lean proteins, and fish have been associated with better subjective sleep evaluations. These findings corroborate those from other studies that investigated the association between diet and sleep quality [30]. Similarly, Theorell-Haglöw et al. (2020) found that reduced sleep duration and poorer sleep quality were linked to less healthy diets and irregular meal timing [31].

Lifestyle factors have been shown to influence dietary patterns, particularly among young adults. In Saudi Arabia, recent research involving university students demonstrated a close association between diet and sleep behaviours [15,17]. Among adults, Alghamdi and Almasaudi (2025) reported similar findings, observing that individuals who consumed more fruits, fish, and legumes had better sleep efficiency, whereas those who consumed more starches, sweets, and dairy products had poorer sleep outcomes [30]. These findings highlight the relationship between dietary behaviour and sleep quality in Middle Eastern populations.

Higher-quality diets, characterised by greater consumption of vegetables, fruits, legumes, nuts, and fish, have been consistently correlated with better sleep outcomes, including longer sleep duration, better sleep efficiency, and improved overall sleep quality [32,33]. Fish consumption, in particular, has been noted for its association with improved sleep outcomes [33]. These dietary components form the foundation of the Healthy Eating Index (HEI). In the current study, higher HEI scores were associated with improved sleep quality, consistent with international evidence. Nutrients obtained from a higher-quality diet support sleep by regulating sleep-related hormones and neurotransmitters and by reducing oxidative stress. Protein-rich foods provide tryptophan, a precursor of serotonin and melatonin, which are crucial for maintaining normal sleep–wake cycles. Additionally, micronutrients such as B vitamins, vitamin C, magnesium, and omega-3 fatty acids support melatonin synthesis and antioxidant defence, enhancing sleep quality [33]. Grandner et al. (2013) reported that abnormal sleep duration was associated with lower consumption of vitamins and minerals, suggesting that the association between sleep quality and nutritional adequacy is bidirectional [20]. This aligns with the current finding that higher diet quality was linked to enhanced sleep.

It is also important to note that individuals with better sleep may be more motivated or health-conscious, which could contribute to healthier dietary behaviours [29,30]. As motivation and health awareness were not assessed in this study, these factors may represent alternative explanations and should be considered in future research. While these mechanisms involving hormones, appetite regulation, and nutrient effects are supported by prior literature, they were not directly measured in the present study. Therefore, these explanations should be considered plausible mechanisms rather than empirical findings of this study [29,30].

In the present study, physical activity was not significantly associated with total diet quality, adequacy, or moderation scores. This suggests that among this group of university students, physical activity alone was not associated with adherence to healthy dietary behaviours [15]. Similarly, research on Saudi adolescents has demonstrated that slightly greater consumption of fruits and vegetables is associated with higher levels of physical activity; however, this association did not result in significantly improved total dietary quality [18]. Findings from other local and international studies on young adults reinforce this observation [34,35]. These university-based studies suggest that although physical activity may promote healthier food choices, total dietary quality is only weakly associated with physical activity among young adults.

Although physical activity was not independently linked to diet quality, it was closely associated with other lifestyle habits, particularly sleep patterns. Sufficient sleep can promote regular exercise, whereas sleep deficiency may result in reduced motivation and energy for physical activity [17,34]. Conversely, regular exercise has been linked to enhanced sleep quality, indicating that the relationship between exercise and sleep may be bidirectional [36]. Thus, while physical activity may not be an independent factor associated with diet quality, the combination of adequate exercise and sufficient sleep tends to have healthier overall lifestyle behaviours.

In this study, BMI was weakly and negatively associated with the adequacy component of diet quality, and in multivariable regression, it was not significantly associated with overall diet quality or moderation. This finding aligns with previous research showing that BMI is not strongly associated with diet quality after accounting for other lifestyle variables [15,37]. The weak association observed in this study may reflect a lower intake of nutrient-rich foods among participants with higher BMI. This is supported by previous research indicating that higher-quality diets are linked to healthier body weights [15,29,37]. These findings suggest that even moderate improvements in dietary behaviour can positively influence weight management in young adults.

While BMI and total sleep quality were not directly associated in the current study, prior studies have demonstrated a link between the two, with higher BMI correlating with poorer sleep outcomes [38] and poor sleep quality correlating with higher BMI [39], suggesting a bidirectional relationship. Sufficient sleep may play a role in regulating appetite-related hormones, improving energy balance, and promoting healthier food choices [29,37]. Interventions promoting better sleep hygiene may be particularly beneficial in university settings, where academic pressures and lifestyle factors can negatively affect sleep.

Although the associations between physical activity, BMI, and diet quality were not robust, this does not diminish their importance in overall health. However, in populations characterised by moderate exercise levels and predominantly normal BMI, improving sleep hygiene may be more effective in promoting healthier dietary behaviours.

This study had several strengths, including a large sample size, the use of validated instruments, and the simultaneous assessment of multiple lifestyle variables. Its external validity was supported by the consistency of its findings with those of previous studies. However, the study also had several limitations. Given the cross-sectional design, causality cannot be inferred. The observed associations between sleep quality and diet may be bidirectional or influenced by unmeasured confounders such as stress, motivation, or health awareness. Additionally, all data on height, weight, dietary intake, physical activity, and sleep were self-reported, which may introduce recall and social desirability biases. The study employed a non-probability snowball sampling technique, resulting in a sample predominantly composed of females and university-educated students. This limits the generalizability of the findings to the broader population of young adults in Saudi Arabia, particularly males and individuals not engaged in higher education. Dichotomization of sHEI and AIS scores may have reduced statistical power. Additionally, the AIS was not fully psychometrically validated in this sample, as the tool is universal and not context- or culture-specific; however, it has since been previously translated and validated in Arabic [40]. Future research should adopt longitudinal designs to better assess causal relationships and incorporate objective measures of physical activity. A more detailed analysis of diet quality subcomponents is recommended to identify the elements most sensitive to sleep quality. Expanding the sample to include participants from other regions would improve generalisability and support the development of culturally tailored interventional strategies.

## 5. Conclusions

Sleep quality emerged as a significant correlate of diet quality across all assessed sHEI domains in this sample of young Saudi adults, whereas no associations were observed for BMI or physical activity. The findings suggest that sleep may represent an important yet often overlooked factor in dietary behaviours. Addressing sleep patterns could enhance the effectiveness of nutrition-related interventions. Further research is warranted to elucidate the causal mechanisms linking sleep quality and diet quality and to assess the psychological factors that may contribute to this relationship.

## Figures and Tables

**Table 1:** Sociodemographic characteristics of the young Saudi adults.

Variables	Category	No.	%
Age (years)	Mean ± SD	21.06 ± 1.92	
	Range	18–25	
Age (group)	<20	110	23
	20–22	260	54.6
	>22	107	22.4
Gender	Male	57	11.9
	Female	421	88.1
Marital status	Married	31	6.5
	Unmarried	447	93.5
Education	Less than high school	2	0.4
	High school	92	19.2
	Diploma	42	8.8
	University	342	71.6
Employment status	Student	396	82.8
	Employed	41	8.6
	Unemployed	41	8.6
Region of residence	Eastern	42	8.8
	Central	270	56.5
	Northern	28	5.9
	Southern	31	6.5
	Western	107	22.3
Total		478	100

**Table 2:** Diet quality and related factors among young Saudi adults.

Variables	Category	No.	%
Diet quality	Good	239	50
	Poor	239	50
Total moderation *	High	259	54.2
	Low	219	45.8
Total adequacy *	High	238	49.8
	Low	240	50.2
Physical activity	High	56	11.7
	Moderate	47	9.8
	Low	375	78.5
Sleep quality	Poor	264	55.2
	Good	214	44.8
BMI	Underweight	89	18.6
	Normal	271	56.7
	Overweight	75	15.7
	Obese	43	9

* Moderation score: Foods to limit for health. Adequacy score: Foods to consume sufficiently for a healthy diet.

**Table 3:** Spearman correlations between adequacy, moderation, and total sHEI scores and lifestyle factors among young Saudi adults.

Variables	Correlation	Adequacy	Moderation	Total sHEI
Age	r	−0.067	0.084	−0.001
	*p*-value	0.141	0.067	0.979
BMI	r	−0.090 *	0.049	−0.039
	*p*-value	0.048	0.289	0.390
Physical activity	r	−0.041	0.082	0.018
	*p*-value	0.376	0.072	0.690
Sleep quality	r	0.059	0.159 **	0.139 **
	*p*-value	0.194	0.000	0.002

* *p*-value ≤ 0.05, ** *p*-value < 0.001.

**Table 4:** Association between adequacy, moderation, and total sHEI scores and lifestyle factors.

		Adequacy Score				Moderation Score				Total sHEI Score			
Variables	Category	Poor	Good	Chi-Square	*p*-Value	Poor	Good	Chi-Square	*p*-Value	Poor	Good	Chi-Square	*p*-Value
BMI	Underweight	48 (20%)	41 (17.2)	1.708	0.43	49 (22.3%)	40 (15.5%)	4.724	0.09	49 (20.5%)	40 (16.7%)	2.045	0.36
	Normal	129 (53.8%)	142 (59.7%)			114 (52.1%)	157 (60.6%)			128 (53.6%)	143 (59.8%)		
	Overweight/obese	63 (26.2%)	55 (23.1%)			56 (25.6%)	62 (23.9%)			62 (26%)	56 (23.5%)		
Physical activity	Low	181 (75.4%)	194 (81.5%)	2.771	0.25	178 (81.2%)	197 (76.1%)	2.402	0.30	188 (78.7)	187 (78.2%)	0.266	0.88
	Moderate	26 (10.8%)	21 (8.8%)			17 (7.8%)	30 (11.6%)			22 (9.2%)	25 (10.5%)		
	High	33 (13.8%)	23 (9.7%)			24 (11%)	32 (12.4%)			29 (12.1%)	27 (11.3%)		
Sleep quality	Poor	143 (59.6%)	121 (50.8%)	3.349	0.67	134 (61.2%)	130 (50.2%)	5.365	0.02	148 (61.9%)	116 (48.5%)	8.131	0.005
	Good	97 (40.4%)	117 (49.2%)			85 (38.8%)	129 (49.8%)			91 (38.1%)	123 (51.5%)		

**Table 5:** Binomial logistic regression of total sHEI and lifestyle factors.

	Total sHEI Score					Adequacy Score					Moderation Score				
Variable	B	S.E.	Wald	*p*	OR	B	S.E.	Wald	*p*	OR	B	S.E.	Wald	*p*	OR
BMI	−0.114	0.113	1.009	0.32	0.892	−0.049	0.113	0.192	0.66	0.952	0.009	0.113	0.007	0.94	1.009
Physical activity	−0.037	0.139	0.070	0.79	0.964	−0.252	0.140	3.235	0.07	0.777	0.111	0.140	0.628	0.42	1.118
Sleep quality	0.555	0.187	8.771	0.003	1.741 *	0.396	0.187	4.495	0.03	1.486 **	0.431	0.187	5.301	0.02	1.539 ***
Constant	−0.509	0.388	1.717	0.19	0.601	−0.141	0.388	0.132	0.72	0.869	−0.622	0.391	2.534	0.11	0.537

* A one-unit increase in sleep quality was associated with a 74.1% increase in the odds of good diet quality. ** A one-unit increase in sleep quality was associated with a 48.6% increase in the odds of good adequacy. *** A one-unit increase in sleep quality was associated with a 53.9% increase in the odds of good moderation.

## Data Availability

Data can be made available upon request.

## Abbreviations

The following abbreviations are used in this manuscript:

AIS	Athens Insomnia Scale
BMI	Body Mass Index
IPAQ	International Physical Activity Questionnaire
sHEI	Short Healthy Eating Index
